# Collagen XVII Promotes Pancreatic Ductal Adenocarcinoma Tumor Growth through Regulation of PIK3R5

**DOI:** 10.1158/2767-9764.CRC-24-0392

**Published:** 2025-08-12

**Authors:** Thomas Hank, Annie Li, Louisa Bolm, Marta Sandini, Taisuke Baba, Natalie Petruch, Julia Strässer, Kenzui Taniue, Jon M. Harrison, Mari Mino-Kenudson, David T. Ting, Keith D. Lillemoe, Andrew L. Warshaw, Carlos Fernández-del Castillo, Kim C. Honselmann, Andrew S. Liss

**Affiliations:** 1 Department of Surgery, Massachusetts General Hospital and Harvard Medical School, Boston, Massachusetts.; 2 Department of General, Visceral and Transplantation Surgery, Heidelberg University Hospital, Heidelberg, Germany.; 3 Department of Pathology, Massachusetts General Hospital and Harvard Medical School, Boston, Massachusetts.; 4 Massachusetts General Cancer Center and Harvard Medical School, Charlestown, Massachusetts.

## Abstract

Pancreatic ductal adenocarcinoma (PDAC) is a highly chemoresistant malignancy with a dismal 11% 5-year survival rate. PDAC tumors are composed of a dense desmoplastic stroma, and the interaction of this collagen-rich tumor microenvironment with PDAC cells promotes their aggressive growth and metastatic spread. In this study, we investigated the role of collagen XVII, a unique transmembrane collagen that connects the extracellular matrix to cytoplasmic signaling complexes. Examination of PDAC tumors revealed that its expression is an independent prognostic biomarker associated with worsened oncological outcomes. Xenograft tumor studies and *in vitro* coculture experiments with PDAC cell lines and immortalized cancer-associated fibroblasts defined a critical role for the tumor microenvironment in mediating the expression of collagen XVII in the cancer compartment of PDAC tumors. Genetic depletion of collagen XVII reduces the viability and migration of PDAC cells *in vitro* and orthotopic tumor formation *in vivo*, underscoring its critical role in PDAC tumor biology. Tumors formed by collagen XVII–depleted PDAC cells exhibit a dramatic downregulation of phosphoinositide 3-kinase regulatory subunit 5, a regulatory subunit of the class I phosphatidylinositol 3-kinase (PI3K) γ complex, and decreased levels of downstream activated AKT. Taken together, these findings reveal collagen XVII signaling as a novel target for PDAC treatment and provide insight into the mechanisms by which it regulates PI3K activity.

**Significance:**

Our study highlights the importance of cross-talk between the tumor microenvironment and PDAC cells in mediating the expression of a key signaling pathway responsible for PI3K activity in PDAC cells.

## Introduction

Pancreatic ductal adenocarcinoma (PDAC) has a poor prognosis and is predicted to become the second leading cause of cancer-related death by 2030 (1, 2). Many of the challenges associated with treating PDAC are due to a highly desmoplastic stroma, primarily composed of abundant fibrosis and cancer-associated fibroblasts (CAF; ref. 3). This pronounced stroma makes up to 80% of the tumor volume, forming a physical barrier that hinders the penetration of chemotherapy drugs and immune cells into the tumor (4). Moreover, the tumor stroma directly affects the behavior of the cancer cells; the interaction of the extracellular matrix (ECM) and CAFs with PDAC cells promotes their aggressive local growth and metastatic dissemination (5).

The underlying genetic alterations contributing to the progression of PDAC have been well characterized. Mutational activation of the KRAS oncogene occurs in >90% of PDAC, and more than half of the tumors inactivate tumor suppressor genes such as *TP53*, *CDKN2A*, and *SMAD4* (6). Although many additional genes are mutated in PDAC, *KRAS* is the only oncogene mutated at high frequency, and its activation triggers a variety of downstream signaling pathways that promote cell growth and migration (7). However, accumulating evidence suggests that additional external signals from the tumor microenvironment are required for the full activation of at least some of these pathways (6, 8).

Recently, the matrisome has gained wide recognition for its contribution to cancer, including PDAC (9–11). Among these ECM and ECM-associated proteins, fibrillar collagens have been extensively characterized, and their varied mechanisms by which they contribute to PDAC cell survival and migration have been defined (12, 13). However, little is known about the contribution of transmembrane collagens to PDAC. Collagen XVII is the largest among this class of collagens and uniquely contains an extensive intracellular domain that connects intracellular cytoplasmic components with the ECM through laminin-5, playing a major role as a cell surface receptor between the intra- and extracellular compartments of stratified or pseudostratified epithelial cells (14, 15). Collagen XVII is best characterized as a crucial component of hemidesmosome type I, together with integrin α6β4, BP230, plectin, and CD151. It forms an anchoring complex that attaches epithelial cells to the underlying basement membrane at the dermal–epidermal junction (16, 17). Although the contribution of collagen XVII to cutaneous homeostasis is well understood, its role in PDAC remains unclear.

In this study, we identified the protumorigenic potential of collagen XVII and revealed its unique function in PDAC biology. Specifically, our data showed that cancer–stroma cross-talk resulted in the transcriptional activation of collagen XVII. Loss of collagen XVII inhibited tumor growth and metastatic dissemination in PDAC, and patients with high collagen XVII levels demonstrated impaired oncological outcomes after pancreatic resection. Importantly, the protumorigenic effects of collagen XVII were regulated through phosphoinositide 3-kinase regulatory subunit 5 (PIK3R5), a regulatory subunit of the phosphatidylinositol 3-kinase γ (PI3Kγ) complex that maintains PI3K-AKT signaling in PDAC. These results highlight collagen XVII as a novel cancer-derived gene in PDAC biology with potential implications for clinical risk stratification and targeted therapies in the future.

## Materials and Methods

### Cell culture

PDAC cell lines MGH609, MGH937, MGH1108, MGH1275, MGH1312, and MGH1319 were established from patient-derived xenograft (PDX) tumors (18). Cell lines were authenticated by comparing their SNPs with the parental PDX tumor lines. Immortalized epithelial cells from normal human pancreatic duct epithelial cells were purchased from ATCC. Immortalized (human telomerase reverse transcriptase) human CAF (CAF-1) cells expressing markers for myofibroblastic CAFs have been described earlier (19). Cells were cultured in DMEM:Nutrient Mixture F-12 (Corning) supplemented with 10% FBS (Atlanta Biological) and 1% penicillin/streptomycin (Gibco). Cells were maintained under standard conditions at 37°C in a humidified atmosphere containing 5% CO_2_. All cell lines were routinely tested for mycoplasma infection using a Venor GeM Mycoplasma Detection Kit (Sigma-Aldrich, MP0025). Coculture experiments with CAF-1 and PDAC cells were performed in a 4:1 ratio with a total of 3 × 10^6^ cells. CAFs stably expressing mCherry and PDAC cells, which were labeled with CellTracker Green CMFDA Dye (Invitrogen), were separated by FACS after 36 hours of coculture. Transwell cocultures of MGH1319 and CAF-1 cells were performed using Transwell six-well plates (Corning) with inserts containing 0.4 μm pores. MGH1319 cells were plated into the wells of the plates, and MGH1319 or CAF-1 cells were plated in the Transwell inserts. After overnight incubation, inserts were placed into wells containing MGH1319 and harvested after 36 hours of coculture.

Individual MISSION lentiviral short hairpin RNAs (shRNA) specifically targeting *COL17A1* (Sigma-Aldrich, SHCLNG-NM_000494) were used for cell infection. The target sequences of the *COL17A1* shRNAs used in this study are shRNA#1: 5′-CCG​GGT​CCA​GTT​CTT​TCG​GAC​TCA​ACT​CGA​GTT​GAG​TCC​GAA​AGA​ACT​GGA​CTT​TTT​G-3′, shRNA#2: 5′-CCG​GCG​AGA​GAG​TGA​AAT​TCT​CGA​GAA​CTC​GAA​TTT​CAC​TCT​CTC​GTT​TTT​G-3′, and shRNA#3: 5′-CCG​GGC​AAT​GGA​CAA​GGA​AGG​AAT​ACT​CGA​GTA​TTC​CTT​CCT​TGT​CCA​TTG​CTT​TTT​G-3′. The sequence of the nontargeting control (NTC) is 5′-CAA​CAA​GAT​GAA​GAG​CAC​CAA-3′. Infected cells were selected with puromycin (Gibco) for 8 to 10 days before experiments were conducted.

### Cell viability assay

For cell viability assays, PDAC cell lines were infected with shRNA specific to *COL17A1* or a control shRNA and selected with puromycin for 8 to 10 days prior to seeding in a 96-well plate at a density of 2,000 cells per well. Cell culture media were replaced after 72 hours, and cell viability was assessed after 5 days by the resazurin reduction assay with the SpectraMax M2 (Molecular Devices).

### Cell migration assays

The *in vitro* wound healing (scratch) assay was performed with PDAC cells expressing NTC or *COL17A1*-specific shRNAs. Cultures of 1.5 × 10^5^ MGH1108, MGH1275, and MGH1319 cells were plated in a 24-well plate and cultured for 16 hours to allow for the formation of confluent cell monolayers. A linear wound was created using a sterile 200 μL pipette tip (USA Scientific). The scratch area was imaged after 0 and 24 hours, and the open scratch area was quantified using Fiji software (RRID:SCR_002285). The transwell cell migration assays were performed with MGH1319 cells expressing NTC or *COL17A1*-specific shRNAs. Cells were cultured overnight in serum-free media containing 1% BSA. Cells (2 × 10^5^; 200 μL) were plated into ThinCert cell culture inserts with 8 μm pores (Greiner Bio-One) that were placed into wells of a 24-well cell culture plate containing 600 μL of serum-free media. After 24 hours, cells were labeled with 8 µmol/L Calcein-AM (MilliporeSigma), and cells that had migrated to the well side of the ThinCert were removed by trypsinization. Cell content was determined by measuring fluorescence using a SpectraMax plate reader. Relative cell migration was normalized to the mean fluorescence of cells expressing NTC.

### Xenograft tumor models

All animal experiments were approved by the Institute of Animal Care and Use Committee of the Partners HealthCare system under protocol (2004N000019). Heterotopic xenograft tumors of MGH1319 were established by subcutaneous injection of PDAC cells (3 × 10^6^ cells) in NOD.Cg-Prkdc^scid^Il2rgt^m1Wjl^/SzJ (NSG) mice (The Jackson Laboratory, RRID:IMSR_JAX:005557). For orthotopic xenograft tumor experiments, PDAC cell lines (MGH1275 and MGH1319) were infected with two shRNAs specific to *COL17A1* or a control shRNA. Cells (1 × 10^6^) suspended in Hank's Balanced Salt Solution containing 1% Matrigel (Corning) were injected into the pancreata of 6-week-old NSG mice. Mice were monitored weekly, and tumor growth was assessed by manual palpation. After appropriate tumor formation was found in the control group, the tumors were harvested, and tumor measurements were determined at necropsy. The metastasis model was conducted by injecting 1 × 10^6^ MGH1319 cells in 100 μL of Hank’s Balanced Salt Solution into the tail vein of NSG mice (*n* = 6 per shRNA). After 5 weeks, the lungs of the mice were processed into formalin-fixed, paraffin-embedded blocks for further analysis.

### Patient cohort

The protocol for this study was reviewed and approved by the Institutional Review Board at Massachusetts General Hospital (2003P001289). A total of 46 patients who underwent upfront resection for PDAC at Massachusetts General Hospital between 2013 and 2017 were identified from a prospectively maintained institutional database, and tissue samples were collected for the construction of a tissue microarray (TMA) with 92 cores. In addition, clinicopathologic data from these individuals were assessed for survival analysis. Follow-up was updated until March 21, 2020, and patients were followed until their last oncological surveillance or until death. In addition, gene expression quantification data and clinical data for pancreatic adenocarcinoma (TCGA-PAAD) were obtained to generate an independent cohort of patients with PDAC with information on survival based on *COL17A1* expression levels.

### IHC

Tissue sections were deparaffinized and rehydrated in TBS. Antigen retrieval was performed with a citrate buffer or a Tris-EDTA pH 9 buffer in a decloaking chamber (Biocare Medical). Tissue sections were blocked with 5% goat serum (Jackson ImmunoResearch) and 0.1% BSA (Fisher Bioreagents) for 1 hour before blocking with avidin and biotin (Vector Laboratories, SP-2001). For single IHC experiments, sections were incubated with the primary antibody specific to collagen XVII (Abcam, ab184996, RRID:AB_3073438), integrin α6 (Abcam, ab181551, RRID:AB_2927695), integrin β4 (Abcam, ab110167, RRID:AB_10866385), laminin-5 (Novus, NBP2-42391, RRID:AB_3306362), Ki-67 (Abcam, ab15580, RRID:AB_443209), α-SMA (Cell Signaling Technology, 19245, RRID:AB_2734735), COX IV (Cell Signaling Technology, 4850, RRID:AB_2085424), pAKT (Cell Signaling Technology, 4060, RRID:AB_2315049), AKT (Cell Signaling Technology, 4685, RRID:AB_2225340), or PIK3R5 (Invitrogen, MA5-26210, RRID:AB_2723482) overnight. Appropriate biotin-labeled secondary antibodies were applied for 1 hour after washing in TBS. Slides were developed using the VECTASTAIN Elite ABC Kit (Vector Laboratories, PK-6100) and the DAB+ Substrate Chromogen System (Dako, K3467). Staining of the hemidesmosome components on TMAs was quantified by an experienced pancreatic pathologist (M. Mino-Kenudson).

For quantitative analysis of cancer cell proliferation, dual IHC experiments were performed. Tissues were stained with antibodies specific to Ki-67 (Abcam, ab15580) and pan-cytokeratin (Santa Cruz Biotechnology, sc-81714) and visualized using an ImmPRESS Duet Double Staining Polymer Kit (Vector Laboratories, MP-7714). The percentage of Ki-67–positive cells was quantified using the manual counting tool by Fiji. For each mouse (*n* = 4 per shRNA), three tissue slides were stained for Ki-67 and pan-cytokeratin, and three images were captured and quantified per tissue slide. For quantification in the metastasis model, lung sections were stained for COX IV. Three images were obtained from three nonconsecutive sections of lung tissue for each mouse (*n* = 6 per shRNA). To determine the COX IV–positive area, Fiji was used to convert the images to eight-bit images. The threshold was manually adjusted to include the total area of the image covered by cells. The threshold was then adjusted to only include COX IV–positive cells, and the difference was measured to determine the percentage of the COX IV–positive area in each image.

### Western blot

Protein lysates were prepared by lysing cells with RIPA buffer [50 mmol/L Tris HCl (pH 8), 150 mmol/L NaCl, 1% NP-40, 0.5% sodium deoxycholate, 0.1% SDS] containing a protease inhibitor (cOmplete Mini Protease Inhibitor Cocktail, Roche, #4693116001). Proteins in lysates were resolved by electrophoresis on 3% to 8% NuPAGE gel systems (Invitrogen) and transferred to a nitrocellulose membrane (GE Healthcare Life Sciences). Membranes were stained with Ponceau S, washed in tris-buffered saline with Tween 20, and blocked in 5% nonfat dried milk in tris buffered saline with Tween 20 for 1 hour before incubation with the primary antibodies specific to collagen XVII (Abcam, ab184996), β-actin (Cell Signaling, S4967S), pAKT (Cell Signaling Technology, 4060), and AKT (Cell Signaling Technology, 4685). Membranes were incubated in horseradish peroxidase–conjugated secondary antibodies (The Jackson Laboratory) for 1 hour and visualized using SuperSignal West Femto (Thermo Fisher Scientific) and the Azure C600 imaging system (Azure Biosystems).

### Gene expression analysis

Total RNA was isolated from cells and tissue using the RNeasy Mini Kit (QIAGEN), and cDNA was prepared using the SuperScript IV First-Strand Synthesis System (Invitrogen, Life Technologies) according to the manufacturer’s instructions. qPCR was performed using TaqMan Gene Expression Master Mix (Applied Biosystems) with the LightCycler 96 System (Roche). TaqMan assays (Applied Biosystems) were employed to evaluate the expression of *COL17A1* (Hs00990046_m1) and *PIK3R5* (Hs01046353_m1). Reactions were normalized by the expression of *β2-microglobulin* (Hs00187842_m1). For RNA sequencing (RNA-seq) analysis, RNA from tumor samples was extracted using the RNeasy Mini Kit (QIAGEN). After library construction, the library was checked with Qubit and real-time PCR for quantification and with a bioanalyzer for size distribution detection. Quantified libraries were pooled and sequenced on NovaSeq PE150 (Novogene). After filtering out the low-quality data, paired-end clean reads were aligned to the reference genome using HISAT2 v2.0.5. By using DESeq2, raw read counts were normalized, and differentially expressed genes (DEG) were identified, in which genes with *q* value <0.05 and log_2_ fold change >2 were considered as DEGs.

### Statistical analysis

All data were analyzed using GraphPad Prism 7.0 software (RRID:SCR_002798). Continuous variables were analyzed using the Student *t* test and one-way ANOVA with Dunnett’s *post hoc* test. Categorical data were analyzed with Fisher’s exact tests. Overall survival was analyzed by the Kaplan–Meier method. The results are shown as mean ± SEM. All statistical tests were two-sided, and statistical significance was accepted at *P* ≤ 0.05.

### Data availability

The RNA-seq data generated in this study are publicly available in the Database of Genotypes and Phenotypes (accession phs003641.v1.p1).

The TCGA-PAAD data that support the findings of this study are available in the NIH Genomic Data Commons Data Portal at phs000178.

The other datasets generated during and/or analyzed during the current study are available from the corresponding author upon reasonable request.

## Results

### Collagen XVII is expressed in PDAC cells and correlates with oncological outcome

**Figure 1:**
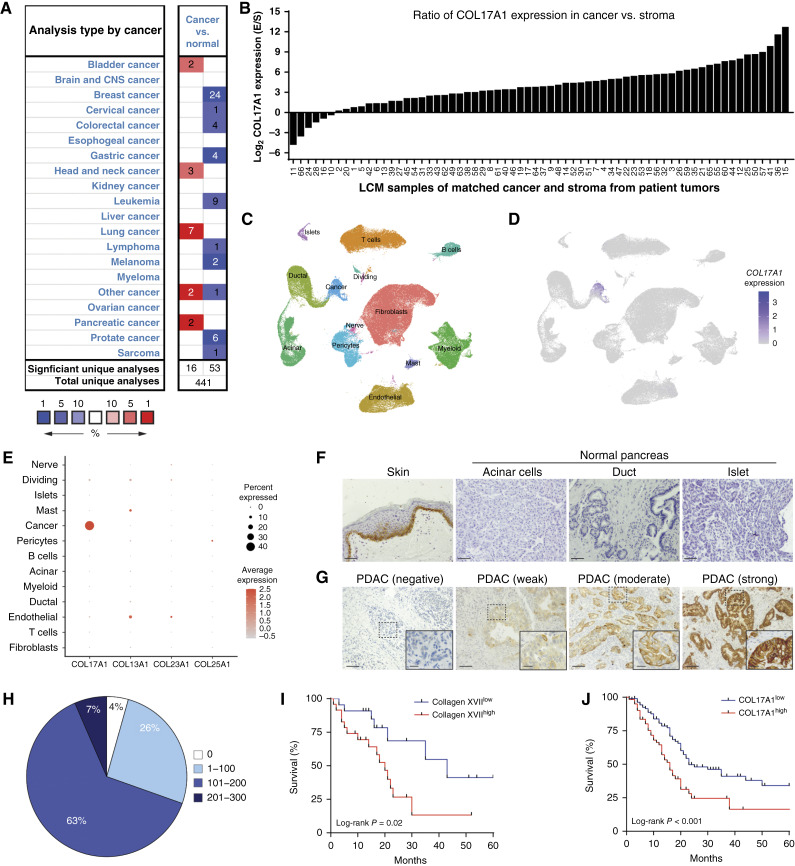
Collagen XVII is expressed in the cancer cells of PDAC tumors and is associated with poor survival. **A,** Analysis of *COL17A1* expression in cancer vs. normal tissues adapted from Oncomine. The numbers in the graphic indicate the number of datasets, grouped by cancer type, showing statistically significant changes in *COL17A1* gene expression between cancer and normal tissues. Significance was defined using the following thresholds: *P* value ≤ 0.05, fold change ≥2, and inclusion in the top 10% of gene expression rankings. Upregulated gene expression is indicated by red cells, and downregulated gene expression is indicated by blue cells. Cell color intensity reflects the best gene rank percentile across all analyses included in the cell. **B,***COL17A1* expression of laser capture microdissected (LCM) separated matched cancer and stroma from tumors of patients with PDAC. Each bar represents the ratio of *COL17A1* expression in a patient sample in cancer compared with the stroma (E/S, epithelial/stroma). **C,** Uniform Manifold Approximation and Projection for Dimension Reduction visualization of single cells from normal pancreas, pancreatitis, and PDAC samples. **D,***COL17A1* expression (purple) in each cell type in the global Uniform Manifold Approximation and Projection for Dimension Reduction. **E,** Bubble plot demonstrating the expression of transmembrane collagens across cell types. The intensity of the color is proportional to the average expression of the gene within a cluster, and the bubble size is proportional to the number of cells expressing the marker. **F,** IHC staining of collagen XVII on skin and acinar cells, ducts, and islets in the normal pancreas. Scale bars, 50 μm. **G,** IHC staining of collagen XVII on PDAC tissue. Representative images show negative, weak, moderate, and strong collagen XVII expression. Scale bars, 100 and 25 µm (inset). **H,***H*-score distribution of collagen XVII expression in 46 treatment-naïve patients with PDAC. **I,** Kaplan–Meier survival analysis of treatment-naïve patients with PDAC for collagen XVII high (*H*-score >130; *n* = 24) vs. low (*H*-score <130; *n* = 22) expressing PDAC tumors (*n* = 46, log-rank test, *P* = 0.02). **J,** Kaplan–Meier survival analysis of patients with PDAC for *COL17A1* high (FPKM >43.77; *n* = 64) vs. low (FPKM ≤43.77; *n* = 112) expressing PDAC tumors (*n* = 176, log-rank test, *P* < 0.001). CNS, central nervous system.

Given the importance of collagen XVII in a variety of epithelial tumors, we sought to explore its role in PDAC. Employing a public dataset of RNA expression from a variety of cancers and normal tissues to investigate the expression pattern of *COL17A1*, the gene encoding collagen XVII, revealed *COL17A1* expression was downregulated in the majority of cancer types relative to normal tissues [Fig. 1A (blue bars)]; however, it was upregualted in several cancer types, including PDAC [Fig. 1A (red bars); ref. 20]. As the dense stromal compartment in PDAC comprises up to 90% of the tumor volume, we sought to clarify the expression pattern of *COL17A1* to further differentiate between cancer or stroma origin (21). We examined *COL17A1* expression in 66 matched pairs of laser capture microdissected cancer and stroma samples generated by Maurer and colleagues (Fig. 1B; ref. 22) and were able to demonstrate a considerably higher expression of *COL17A1* in the cancer compartment in 55 of 66 samples (83.3%). A more detailed analysis of cells that express *COL17A1* was performed using single-cell RNA-seq results from 19 samples of normal pancreas, pancreatitis, and PDAC (Fig. 1C), which found that it was expressed almost exclusively in the cancer cells (Fig. 1D; ref. 23). Moreover, the expression of *COL17A1* in PDAC cells was unique among transmembrane collagens, with only limited expression of *COL13A1*, *COL23A1*, and *COL25A1* observed in a subset of stromal cells (Fig. 1E).

With this information, we performed IHC staining for collagen XVII. As shown in Fig. 1F (left), human skin exhibits robust membranous staining for collagen XVII, which served as a positive control. In contrast, collagen XVII was not detected in normal pancreatic tissues derived from human organ donors, including acinar cells, ductal epithelial cells, and islets (Fig. 1F). To elucidate the expression of collagen XVII in PDAC samples, we employed a TMA composed of 92 cores of PDAC from 46 treatment-naïve patient tumors. These studies revealed a heterogeneous expression of collagen XVII (Fig. 1G), which was quantified using the *H*-score. By applying four intensity levels, nearly 70% of patients exhibited strong (*H*-score 101–200; 63.04%) to very strong (*H*-score 201–300; 6.5%) positivity for collagen XVII (Fig. 1H), and only one tumor sample was negative for collagen XVII expression. Consistent with the RNA expression studies, we found that collagen XVII was limited to cancer cells, with the stromal compartment nearly devoid of collagen XVII expression.

Based on the broad expression of collagen XVII in PDAC tumors, we investigated whether these expression profiles were associated with oncological outcomes in our patient cohort (Supplementary Table S1). Kaplan–Meier survival analysis (Fig. 1I) revealed that patients with high collagen XVII expression (*H*-score >130) had a worse outcome compared with patients with weak expression of collagen XVII, with a median overall survival of 20 versus 43 months, respectively (*P* = 0.02). A similar analysis using The Cancer Genome Atlas dataset was conducted for external validation (Fig. 1J). Examining *COL17A1* RNA expression levels from 177 resected patients with PDAC revealed decreased survival in patients with higher *COL17A1* levels (median: 91.5 FPKM) compared with individuals with lower *COL17A1* levels (median: 10.95 FPKM), with a median survival of 16 versus 23 months, respectively (*P* < 0.003). Interestingly, although *COL17A1* was broadly expressed across tumors, classical subtype tumors were more prevalent in the high *COL17A1* group (*P* < 0.001), whereas nearly equivalent numbers of classical and basal-like tumors were found in the low *COL17A1* group (Supplementary Table S2). Taken together, these results demonstrate that collagen XVII expression is induced in PDAC cells, and its levels correlate with oncological outcomes, suggesting a key role for collagen XVII in pancreatic tumor biology.

### Profiling of associated hemidesmosome and ECM components in PDAC

**Figure 2:**
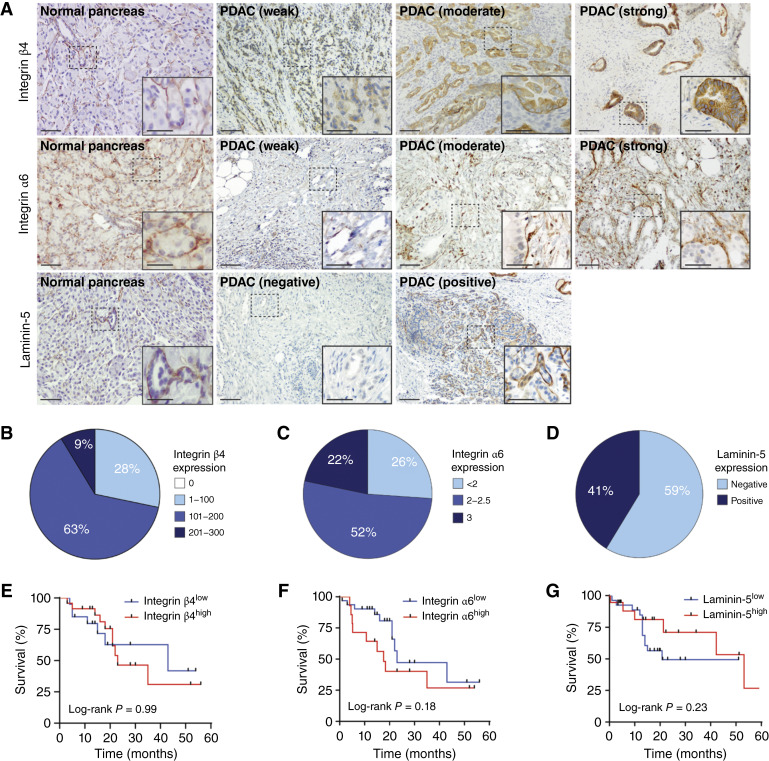
Hemidesmosome components integrin β4, integrin α6, and laminin-5 are not correlated with survival in patients with PDAC. **A,** Representative images of IHC staining of integrin β4, integrin α6, and laminin-5 in normal pancreas and PDAC tissues from 46 treatment-naïve patients with PDAC. Scale bars, 100 and 25 µm (inset). **B,***H*-score distribution of integrin β4 expression. **C,** Distribution of integrin α6 expression scores ranging from 1 to 3. **D,** Percentage of PDAC samples positive and negative for laminin-5. **E,** Kaplan–Meier survival analysis of high (*H*-score >131; *n* = 24) vs. low (*H*-score <131; *n* = 22) integrin β4–expressing patients with PDAC. **F,** Kaplan–Meier survival analysis of high (score >2; *n* = 14) vs. low (score ≤2; *n* = 32) integrin α6–expressing patients with PDAC. **G,** Kaplan–Meier survival analysis of positive (*n* = 19) vs. negative (*n* = 27) laminin-5–expressing patients with PDAC.

Collagen XVII is the defining component in the formation of type I hemidesmosome complexes in (pseudo) stratified epithelium. We, therefore, sought to determine the expression levels of additional hemidesmosome components (integrin β4 and integrin α6) and an associated ECM protein (laminin-5). In contrast to collagen XVII, these proteins are also found in hemidesmosome type II, which is present in simple epithelia such as the pancreas (16). Consequently, we were able to detect all components in normal pancreatic tissue [Fig. 2A (left column)]. Staining for integrin β4, integrin α6, and laminin-5 demonstrated membranous expression in ductal and acinar cells.

Next, we employed our established TMA to investigate the expression profiles of the associated members in malignant tissue. In line with their broad expression in normal pancreatic tissue, integrin α6/β4 and laminin-5 showed heterogeneous expression patterns in the basement membrane of malignant epithelial cells (Fig. 2A–D). We then examined whether the expression levels of these components were associated with oncological outcomes in PDAC by Kaplan–Meier analysis. In contrast to collagen XVII, the expression of these hemidesmosome components did not correlate with overall survival in our cohort of patients with PDAC (Fig. 2E–G). With the broad expression of type II hemidesmosome components in the normal pancreas, these results suggest that the *de novo* expression of collagen XVII results in the formation of the type I hemidesmosome in PDAC. However, we did not observe a significant correlation between the expression levels of collagen XVII and integrin α6/β4 or laminin-5, so a hemidesmosome-independent role for collagen XVII cannot be excluded (Supplementary Fig. S1).

### Cancer–stroma cross-talk induces collagen XVII expression

**Figure 3:**
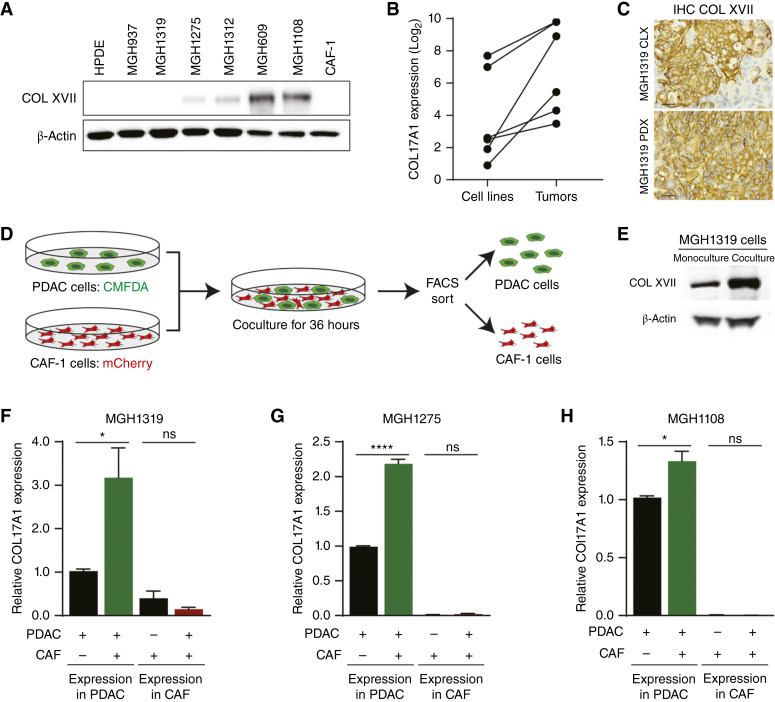
Collagen XVII expression is upregulated in PDAC cells upon interaction with CAFs. **A,** Western blot analysis of collagen XVII expression in immortalized human pancreatic ductal cells [human pancreatic duct epithelial (HPDE)], six PDAC cell lines, and telomerase reverse transcriptase–immortalized CAFs (CAF-1). β-Actin is shown as a loading control. **B,***COL17A1* expression in cell lines and matching xenograft tumors. **C,** Representative IHC staining of collagen XVII in cell line xenografts and PDXs of MGH1319. Scale bars, 50 μm. **D,** Schematic of the experimental setup of coculture and FACS of PDAC and CAF-1 cells. **E,** Collagen XVII expression in MGH1319 cells in monoculture and after coculture with CAF-1. β-Actin is shown as a loading control. **F–H,** Relative *COL17A1* expression in MGH1319, MGH1275, MGH1108, and CAF-1 after mono- and coculture. The average (±SEM) from three independent experiments is shown. *, *P* < 0.05; ****, *P* < 0.0001; ns, not signficant (unpaired *t* test).

To begin to interrogate the role of collagen XVII in PDAC biology, collagen XVII levels were determined in human PDAC cell lines by Western blot analyses (Fig. 3A). Collagen XVII is heterogeneously expressed among our cell lines established from PDX tumors, with cell lines expressing low (MGH937, MGH1319), moderate (MGH1275, MGH1312), or high (MGH609, MGH1108) levels of collagen XVII. Consistent with the absence of collagen XVII in normal pancreas and tumor stroma, collagen XVII was not detected in cell lysates from immortalized ductal epithelial cells (human pancreatic duct epithelial cells) and CAFs (CAF-1). Interestingly, examining the RNA from these cell lines and their parental PDX models revealed that the PDX models expressed up to fourfold higher levels of *COL17A1* (Fig. 3B). Importantly, the implantation of cell lines with low levels of collagen XVII into mice resulted in xenograft tumors with high levels of collagen XVII that were comparable with the parental PDX tumor from which the cell line was derived (Fig. 3C). Taken together, these results suggest that the tumor microenvironment stimulates *COL17A1* expression.

To further investigate the contribution of cancer–stroma cross-talk to *COL17A1* expression, *in vitro* experiments were conducted employing mono- and cocultures of fluorescently labeled (CMFDA) PDAC cells and CAF-1 cells constitutively expressing mCherry (Fig. 3D). The PDAC and CAF-1 cells underwent FACS and were analyzed for the expression of collagen XVII protein and RNA. The expression of collagen XVII was dramatically induced in MGH1319 cells when cocultured with CAF-1 cells (Fig. 3E). This increase in collagen XVII protein correlated with an increase in *COL17A1* RNA levels in MGH1319 cells (Fig. 3F). MGH1275 and MGH1108 cells also exhibited an upregulation of *COL17A1* when cocultured with CAF-1 cells (Fig. 3G and H). Notably, the extent of the upregulation was inversely proportional to levels of *COL17A1* in the monocultures. As a result, MGH1319 demonstrated the strongest upregulation of *COL17A1* with a more than threefold increase in comparison with the baseline expression [*P* < 0.05; Fig. 3F (left)]. Moreover, only low levels of *COL17A1* were detected in CAF-1 cells and were unaltered by the addition of cancer cells. Coculturing CAF-1 cells with MGH1319 cells using a transwell cell culture system that prevented direct contact between these cell populations did not result in an upregulation of *COL17A1*, suggesting that physical contact between PDAC cells and CAFs mediates the CAF-induced expression of *COL17A1* (Supplementary Fig. S2). Together, these findings highlight that the expression of *COL17A1* in PDAC cancer cells is enhanced by human CAFs *in vitro* and murine stroma *in vivo*, highlighting the role of cancer–stroma cross-talk in regulating *COL17A1*.

### Loss of collagen XVII reduces tumor cell viability and metastatic potential

**Figure 4:**
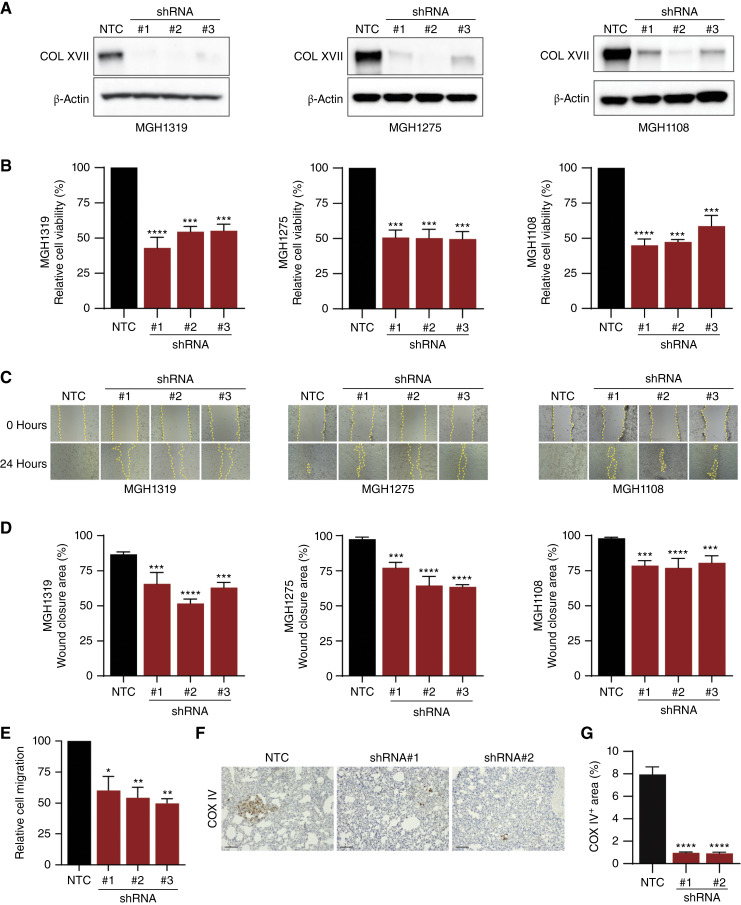
Viability and metastatic potential of PDAC cells is dependent on collagen XVII. **A,** Western blot analysis of collagen XVII in MGH1319, MGH1275, and MGH1108 cells expressing an NTC shRNA and *COL17A1*-specific shRNAs. The expression of β-actin served as a control. **B,** Resazurin-based cell viability assays were performed using MGH1319, MGH1275, and MGH1108 cells expressing NTC and *COL17A1*-specific shRNAs. The mean (±SEM) cell viability relative to NTC-expressing cells of three independent experiments performed in triplicate is shown. ***, *P* < 0.001 (one-way ANOVA with Dunnett’s *post hoc* test). **C,** Representative scratch areas at 0 and 24 hours of MGH1319, MGH1275, and MGH1108 cells expressing NTC and *COL17A1*-specific shRNAs. **D,** The mean (±SEM) scratch closure area relative to 0 hours from three independent biological experiments is shown for MGH1319, MGH1275, and MGH1108. ***, *P* < 0.001; ****, *P* < 0.0001 (one-way ANOVA with Dunnett’s *post hoc* test). **E,** The mean (±SEM) migration of MGH1319 cells expressing *COL17A1*-specific shRNAs relative to cells expressing NTC, as measured by a transwell migration assay. Three independent infections of MGH1319 are shown. *, *P* < 0.02; **, *P* < 0.006 (one-way ANOVA with Dunnett’s *post hoc* test). **F,** Representative images of COX IV IHC staining of lungs harvested from mice 4 weeks after injection with MGH1319 cells expressing an NTC vector or *COL17A1*-specific shRNAs. Scale bars, 100 µm. **G,** Percentage of COX IV–positive area (±SEM) in the lungs of mice with metastasis formed by MGH1319 cells expressing either an NTC or *COL17A1*-specific shRNAs (*n* = 6 for each shRNA). ****, *P* < 0.0001 (one-way ANOVA with Dunnett’s *post hoc* test).

To examine the contribution of collagen XVII to PDAC, we established three lentiviral-mediated shRNAs to suppress collagen XVII expression in MGH1319, MGH1275, and MGH1108 cells (Fig. 4A). Resazurin-based assays revealed a 40% to 50% reduction in cell viability after suppression of collagen XVII relative to cells expressing an NTC shRNA (*P* < 0.001; Fig. 4B). These effects were observed across cell lines with different baseline expressions of collagen XVII (compare Figs. 3A and 4B), indicating that even low levels of collagen XVII contribute to PDAC cell growth *in vitro*.

Next, we performed scratch assays to investigate additional biological functions of collagen XVII (Fig. 4C). We observed reduced cell migration in PDAC cells with diminished collagen XVII levels in comparison with control cells for all three cell lines. By 24 hours, control cells exhibited nearly complete wound closure, whereas PDAC cells suppressing collagen XVII had significantly less closure [MGH1319: 86% vs. 66% (*P* < 0.001), 52% (*P* < 0.0001), and 64% (*P* < 0.0001); MGH1275: 98% vs. 77% (*P* < 0.001), 64% (*P* < 0.0001), and 63% (*P* < 0.0001); MGH1108: 98% vs. 79% (*P* < 0.001), 77% (*P* < 0.0001), and 81% (*P* < 0.001); Fig. 4D]. A similar reduction in cell migration was observed in MGH1319 cells with diminished collagen XVII levels using a transwell cell migration assay (Fig. 4E), suggesting an important role for collagen XVII in the migratory ability of PDAC cells.

To examine whether these *in vitro* effects resulting from the loss of collagen XVII affected PDAC cells *in vivo*, we employed a murine metastasis model that examines the terminal steps of metastasis, extravasation from the circulatory system, and establishment of metastatic foci. MGH1319 cells with or without suppression of collagen XVII were implanted into NSG mice by tail vein injection, and after 3 weeks, the lungs of the mice were harvested for analysis of pulmonary metastasis. To assess the extent of metastasis, lung sections were stained with a monoclonal antibody that recognizes human, but not mouse, COX IV to detect the human PDAC cells in the context of the murine lung (Fig. 4F). Quantification of the COX IV–stained area revealed a more than sevenfold decrease in COX IV–positive cells in the lungs of mice implanted with *COL17A1*-deficient MGH1319 cells relative to mice implanted with NTC-expressing cells (*P* < 0.0001; Fig. 4G). Together, these results demonstrate that not only does collagen XVII play a role in the growth and migration of PDAC cells *in vitro*, but it is also a key contributor to the metastatic dissemination of PDAC cells *in vivo*.

### Collagen XVII affects tumor growth *in vivo*

**Figure 5:**
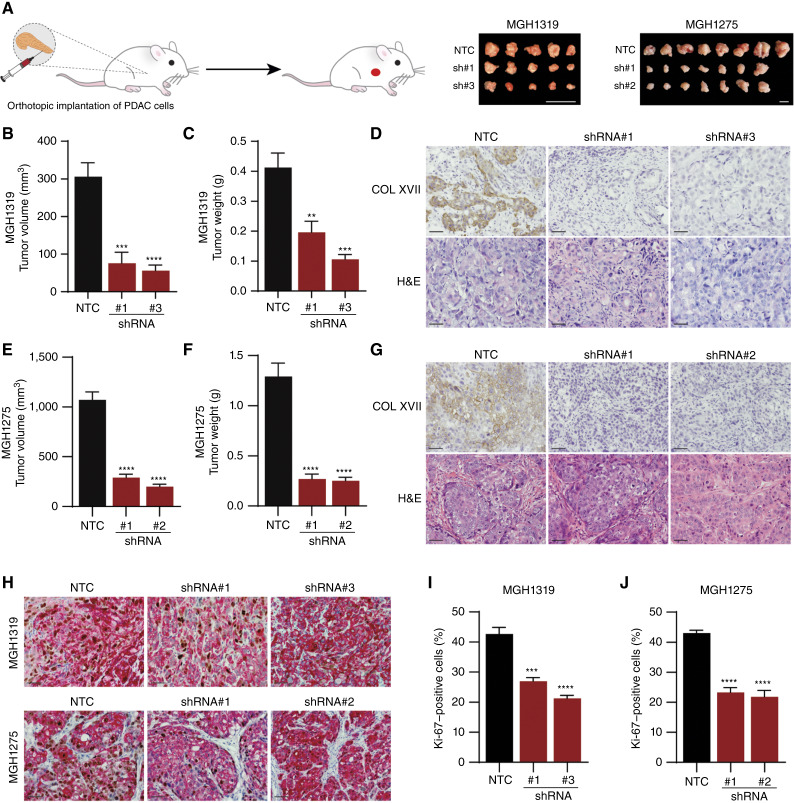
Reduced tumor forming potential of *COL17A1*-deficient PDAC cells. **A,** Schematic of orthotopic implantations of PDAC cells into the pancreata of NSG mice and photographs of orthotopic tumors formed by MGH1319 and MGH1275 cells expressing NTC and *COL17A1*-specific shRNAs. Scale bars, 1 cm. **B** and **C,** MGH1319 expressing NTC and *COL17A1*-specific shRNAs were implanted into the pancreas of mice. Tumor volumes were calculated by caliper measurement (**B**), and mass was determined (**C**). Data are presented as mean (±SEM) from five mice per group. **, *P* < 0.01; ***, *P* < 0.001; ****, *P* < 0.0001 (one-way ANOVA with Dunnett’s *post hoc* test). **D,** Representative images of collagen XVII IHC (top) and hematoxylin and eosin (H&E) staining (bottom). Scale bars, 50 µm. **E** and **F,** MGH1275 cells expressing NTC and *COL17A1*-specific shRNAs were implanted into the pancreas of mice. Tumor volumes were calculated by caliper measurement (**E**) and mass determined (**F**). Data are presented as mean (±SEM) from five mice per group. ****, *P* < 0.0001 (one-way ANOVA with Dunnett’s *post hoc* test). **G,** Representative images of collagen XVII IHC (top) and H&E staining (bottom). Scale bars, 50 µm. **H,** IHC staining of pan-cytokeratin (red) and Ki-67 (brown). **I** and **J,** Percentage of Ki-67–positive cancer cells in tumors formed by MGH1319 cells (**I**) and MGH1275 cells (**J**) expressing NTC and *COL17A1*-specific shRNAs. The average (±SEM) from four tumors is shown. ***, *P* < 0.001; ****, *P* < 0.0001 (one-way ANOVA with Dunnett’s *post hoc* test).

To further examine the effects of collagen XVII loss in PDAC, we performed orthotopic xenograft tumor experiments in NSG mice. Independent experiments with MGH1319 and MGH1275 cells were conducted after suppression of *COL17A1* by two shRNAs, as well as with cells expressing the control NTC. Tumors formed by *COL17A1*-deficient MGH1319 cells were smaller in comparison with NTC tumors 5 weeks after implantation in the pancreas of mice (Fig. 5A). Quantification of the tumors showed that depletion of *COL17A1* by shRNA#1 and shRNA#3 reduced tumor weight by 52% and 74% and tumor size by 75% and 82%, respectively (*P* < 0.01; Fig. 5B and C). Similarly, tumors formed by *COL17A1*-deficient MGH1275 cells were significantly smaller compared with NTC tumors, with a reduction in tumor weight of 79% and 81% and a reduction in size of 73% and 81% for shRNA#1 and shRNA#2, respectively (*P* < 0.0001; Fig. 5A, E, and F).

IHC staining for collagen XVII in the tumor samples revealed the continuous suppression of collagen XVII in both xenograft tumor models, suggesting that the growth differences were due to a reduced tumor-forming potential of these cells as opposed to a selection of a subpopulation of cells that maintained collagen XVII levels (Fig. 5D and G). Consistent with this, Ki-67 staining revealed a reduction in the proliferation index in collagen XVII–deficient tumors in contrast to control NTC-expressing tumors (Fig. 5 H–J). IHC staining for cleaved caspase-3 did not reveal appreciable levels of apoptotic cells in control tumors or those with diminished collagen XVII. Moreover, similar levels of collagen and α-SMA–expressing cells were observed in the tumor stroma (Supplementary Fig. S3), suggesting that the reduced proliferation of the cancer cells was mainly responsible for the reduced tumor growth.

### Collagen XVII mediates PI3K/AKT signaling in PDAC via PIK3R5

**Figure 6:**
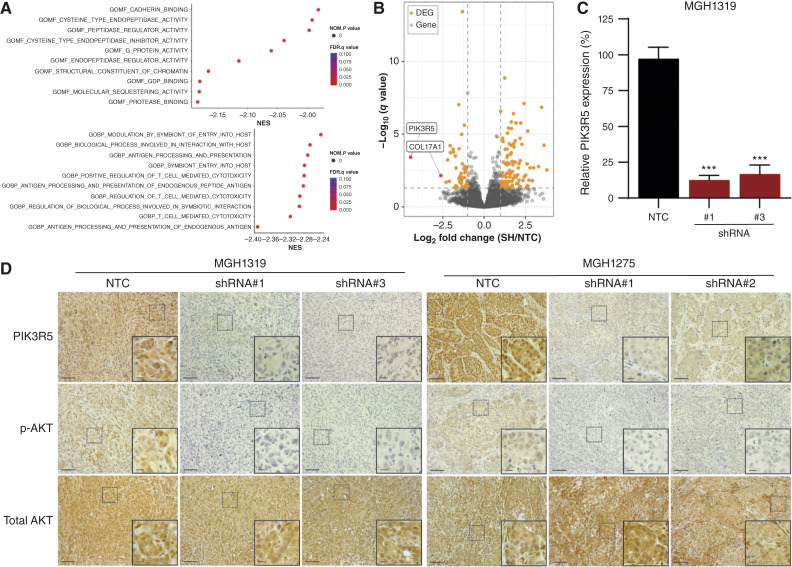
The expression of PIK3R5 and activation of AKT is dependent on collagen XVII. **A,** Bubble plots illustrating two upregulated gene ontology programs in collagen XVII–deficient MGH1275 cells. **B,** Volcano plot showing DEGs in tumors formed by MGH1275 cells expressing an NTC vector vs. *COL17A1*-specific shRNAs. **C,** Relative expression of *PIK3R5* in tumors formed by MGH1319 cells expressing an NTC shRNA and *COL17A1*-specific shRNAs. The mean (±SEM) from three tumors analyzed in triplicate is shown. ***, *P* < 0.001, one-way ANOVA with Dunnett’s *post hoc* test. **D,** IHC staining of PIK3R5, P-AKT, and total AKT on orthotopic tumors formed by MGH1319 and MGH1275 cells expressing an NTC vector or *COL17A1*-specific shRNAs. Scale bars, 100 and 25 µm (inset). FDR *q* value, Fasle Discovery Rate-adjusted *q*-value; NES, normalized enrichment scale; NOM_*P* value, nominal *P*-value.

The *in vivo* experiments above provide strong evidence for the importance of collagen XVII in PDAC tumor growth, which prompted us to interrogate the relevant mechanisms by which collagen XVII contributes to PDAC biology. Toward this end, we performed RNA-seq on tumors formed by MGH1275 cells expressing NTC or *COL17A1*-specific shRNAs. Gene ontology analysis revealed an upregulation of genes involved in endopeptidase activity, antigen presentation, and T cell–mediated cytotoxicity (Fig. 6A). More than three quarters of the top 40 most DEGs were upregulated upon loss of *COL17A* (Supplementary Fig. S4A). Consistent with the role of collagen XVII in the epidermis, a number of these differentially expressed genes are involved in skin homeostasis (*SPINKK6*, *SPRR1A*, *TGM3*, *IVL*). Among the most dramatically downregulated genes in *COL17A1*-deficient tumors was *PIK3R5* (Fig. 6B). A decreased expression of *PIK3R5* was also observed in MGH1319 tumors with diminished collagen XVII levels (Fig. 6C). Importantly, IHC for PIK3R5 in both MGH1319 and MGH1275 xenograft tumors revealed a dramatic decrease in its expression in collagen XVII–deficient tumors [Fig. 6D (top row)], suggesting PIK3R5 is a conserved downstream effector of collagen XVII in PDAC.

*PIK3R5* encodes the p101 regulatory subunit of PI3Kγ, which activates PI3K/AKT signaling (24). As PI3K/AKT signaling is critical for PDAC growth, we examined whether this pathway is altered in tumors deficient in collagen XVII. Strikingly, IHC for active, phosphorylated AKT revealed a dramatic loss of its expression without altering total levels of AKT [Fig. 6D (middle and lower)]. A similar downregulation of phosphorylated AKT was seen with *in vitro* cultured MGH1319 and MGH1275 with diminished expression of collagen XVII (Supplementary Fig. S4B). Although experiments directly evaluating the function of PIK3R5 remain to be performed, these results support the notion that collagen XVII mediates the activity of PI3K signaling in tumors through activation of one of its regulatory subunits (p101).

## Discussion

Beyond its role in maintaining the stability of epithelial cells as a component of the hemidesmosome type I, there is a growing recognition of the role of collagen XVII in cancer. In this study, we present evidence for the involvement of collagen XVII in PDAC, indicating that its expression is associated with impaired oncological survival and acts as an independent prognostic biomarker. Although collagen XVII is normally absent in pancreatic tissue, its broad expression in PDAC cells highlights an extracutaneous role for this protein. Mechanistically, collagen XVII promotes the expression of PIK3R5, a subunit of the PI3Kγ complex that activates PI3K/AKT signaling in PDAC, thereby suppressing cell migration and tumor growth. Our metastasis model further supports a role for collagen XVII in the metastatic dissemination of PDAC cells. However, the impact of collagen XVII depletion on PDAC cell viability and tumor growth prevents us from understanding what stage of the metastatic process is dependent on collagen XVII. Nevertheless, these findings underscore the novel role of collagen XVII as a driver in human PDAC.

One major finding of this study is the crucial role of cancer–stroma cross-talk in the expression of collagen XVII in PDAC. A robust desmoplastic stroma is a hallmark of PDAC and has been implicated in the chemoresistance and aggressive behavior of this disease. In our previous work, we showed that cross-talk between PDAC cells and CAFs induces broad transcriptional changes in PDAC, with many of these genes encoding components of the matrisome 9. Interestingly, *COL17A1* was among our 65-gene matrisome signature that predicts oncologic outcomes. In the present study, we found that collagen XVII was exclusively expressed in the cancer compartment, with CAFs playing a critical role in its upregulation. This effect was most pronounced in cell lines with low expression of *COL17A1*, and a similar induction was observed when PDAC cells were grown as xenograft tumors with a murine stroma. Our data suggest that collagen XVII could be a novel target in the treatment of PDAC, as it is a cancer-specific marker and is upregulated as a result of cancer–stroma cross-talk.

There is evidence that collagen XVII plays a role in other intestinal tumors, revealing its role as a cancer-derived gene although physiologically absent in the surfaces arising from simple epithelial cells. In colorectal cancer, elevated protein levels of collagen XVII were linked to more advanced tumor–node–metastasis stages (25). In addition, the authors showed a colocalization of collagen XVII together with its binding partner laminin-5 to promote tumor budding and an infiltrative growth pattern (26). Another study focusing on tumor-initiating cells found increased levels of *COL17A1* in a STAT-3–dependent manner, which correlated inversely with survival in patients with colorectal cancer (27). Strikingly, blockade of this pathway decreased the survival of tumor-initiating cells and abrogated tumor formation in mice. Similar findings were reported for lung cancer, in which higher collagen XVII levels were associated with poor prognosis (28, 29). Moreover, collagen XVII promotes epithelial-to-mesenchymal transition and increased metastatic spread in lung cancer through regulation of the FAK/AKT/GSK3β pathway (28).

Notably, our study identifies a novel mechanism by which collagen XVII regulates the PI3K/AKT pathway through the regulation of PIK3R5. PIK3R5 encodes the p101 regulatory subunit, which interacts with PI3K by recruiting the catalytic subunit class IB (p110γ; ref. 30). PI3Ks generate the second messenger phosphatidylinositol 3,4,5-trisphosphate, which activates multiple downstream pathways (e.g., AKT) and are divided into three different classes, of which only class I enzymes are known to be involved in oncogenesis (31). The PI3K/AKT signaling pathway is recognized as playing an important role in cell survival, cell growth, and tumorigenesis (32). In PDAC, it promotes the survival of cancer cells by blocking proapoptotic proteins while also enhancing their mobility and metastatic abilities (24). PIK3R5 plays an important role in cell growth and cell motility and has been found to be overexpressed in ovarian cancer that shows high levels of chemoresistance, which is a common feature in PDAC (30, 33). Consequently, these findings highlight the importance of collagen XVII in maintaining PI3K/AKT signaling in PDAC via PIK3R5.

Taken together, our study identifies collagen XVII as a cancer-specific protumorigenic protein in PDAC, which has significant implications for the biology and survival of patients with the disease. Through upregulation by cancer–stroma cross-talk, collagen XVII promotes tumor growth and metastatic dissemination. Importantly, patients with high expression of collagen XVII have a worse oncological outcome, highlighting its clinical relevance. Mechanistically, collagen XVII interacts with the PI3K/AKT pathway via PIK3R5, indicating the potential of collagen XVII signaling as a novel target in the multidimensional treatment of PDAC.

## Supplementary Material

Supplementary Table S1 Patient characteristics of tumors used immunohistochemical analysis of hemidesmosome components

Supplementary Table S2 Patient characteristics of tumors used for RNA-seq analysis of hemidesmosome components

Supplemental Figure S1 Plots correlating expression of COLXVII with other hemidesmosome components

Supplemental Figure S2 Expression of COL17A1 after Trasnwell co-culture of PDAC cells and CAFs

Supplemental Figure S3 Immunohistochemical and histologic analysis of collagen and fibroblast content of tumors formed by COLXVII-deficient PDAC cells

Supplemental Figure S4 Changes in gene expression and active AKT levels in collagen XVII-deficient cells
