# Supplementary material for: Collagen XVII Promotes Pancreatic Ductal Adenocarcinoma Tumor Growth through Regulation of PIK3R5
Source: Cancer Res Commun. 2025 Aug 12;5(8):1319–31. doi: 10.1158/2767-9764.CRC-24-0392 (PMC12340215; doi:10.1158/2767-9764.CRC-24-0392)
Supplement: Supplementary Table S1 — Patient characteristics of tumors used immunohistochemical analysis of hemidesmosome components [file crc-24-0392_supplementary_table_s1_suppst1.docx]

|  | **Collagen XVII low (n=22)** | **Collagen XVII high (n=24)** | **p-value** |
| --- | --- | --- | --- |
| **Gender M/F** | 12/10 (54.5/45.5) | 11/13 (45.8/54.2) | 0.555 |
| **Age (years)** | 66±12 | 73±9 | 0.026 |
| **Tumor size (mm)** | 31±13 | 34±20 | 0.489 |
| **T-Stage**  **1**  **2**  **3** | 4 (18.2)  13 (59.1)  5 (22.7) | 3 (12.5)  14 (58.3)  7 (29.2) | 0.808 |
| **N-Stage**  **0**  **1**  **2** | 6 (27.3)  8 (36.4)  8 (36.4) | 9 (37.5)  8 (33.3)  7 (29.2) | 0.748 |
| **Grading**  **G1**  **G2**  **G3** | 5 (22.7)  11 (50.0)  5 (22.7) | 2 (8.3)  10 (41.7)  11 (45.8) | 0.174 |
| **Neural invasion**  **0**  **1** | 3 (13.6)  19 (86.4) | 3 (12.5)  21 (87.5) | 0.909 |
| **Vascular Invasion**  **0**  **1** | 12 (54.5)  10 (45.5) | 9 (37.5)  15 (62.5) | 0.246 |
| **R-status**  **0**  **1** | 13 (59.1)  9 (40.9) | 20 (83.3)  4 (16.7) | 0.068 |
| **Adjuvant treatment**  **0**  **1** | 5 (22.7)  17 (77.3) | 9 (37.5)  15 (62.5) | 0.277 |

**Supplementary Table S1: Patient characteristics of MGH cohort**
