# Supplementary material for: Collagen XVII Promotes Pancreatic Ductal Adenocarcinoma Tumor Growth through Regulation of PIK3R5
Source: Cancer Res Commun. 2025 Aug 12;5(8):1319–31. doi: 10.1158/2767-9764.CRC-24-0392 (PMC12340215; doi:10.1158/2767-9764.CRC-24-0392)
Supplement: Supplementary Table S2 — Patient characteristics of tumors used for RNA-seq analysis of hemidesmosome components [file crc-24-0392_supplementary_table_s2_suppst2.docx]

**Supplementary Table S2: Patient characteristics of TCGA cohort**

|  | **COL17A1 low n=89** | **COL17A1 high n=88** | p-value |
| --- | --- | --- | --- |
| **Gender M/F** | 41/48 (46.1/53.9) | 57/32 (64.7/35.3) | 0.018 |
| **AJCC Stage**  **I-II**  **III-IV** | 22  66 | 9  80 | 1.0 |
| **T stage**  **1**  **2**  **3** | 4  13  5 | 3  13  8 | 0.829 |
| **N stage**  **0**  **1+** | 27  59 | 27  63 | 1.0 |
| **M stage**  **0**  **1** | 88  1 | 85  3 | 0.367 |
| **Grading**  **G1**  **G2**  **G3** | 5  33  24 | 2  42  45 | 1.0 |
| **Molecular subtype**  **Classical**  **Basal-like** | 26  27 | 50  36 | <0.001 |
