# Supplementary figures and images for: Collagen XVII Promotes Pancreatic Ductal Adenocarcinoma Tumor Growth through Regulation of PIK3R5
Source: Cancer Res Commun. 2025 Aug 12;5(8):1319–31. doi: 10.1158/2767-9764.CRC-24-0392 (PMC12340215; doi:10.1158/2767-9764.CRC-24-0392)

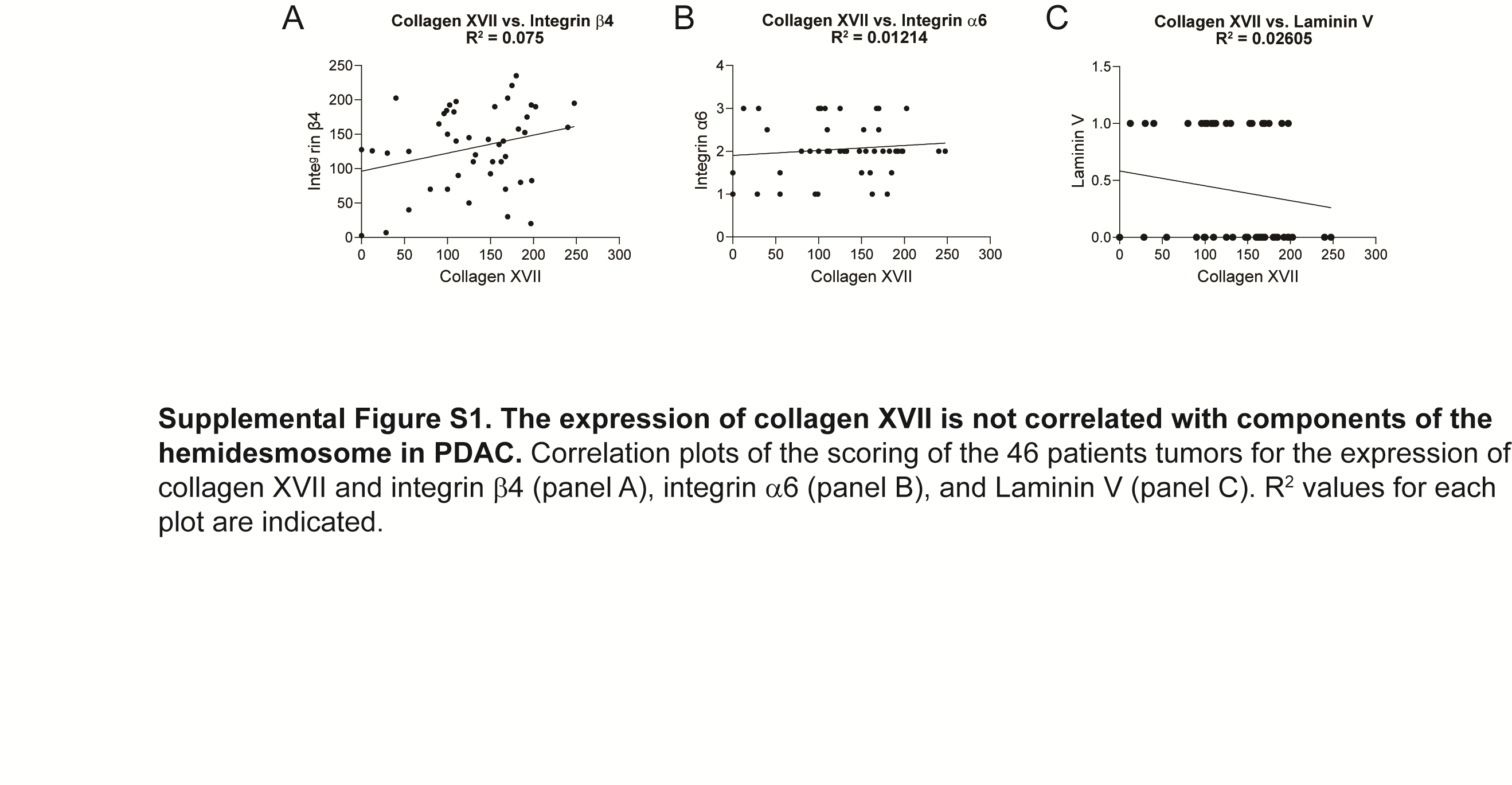

Supplement: Supplemental Figure S1 — Plots correlating expression of COLXVII with other hemidesmosome components [file crc-24-0392_supplemental_figure_s1_suppsf1.png]

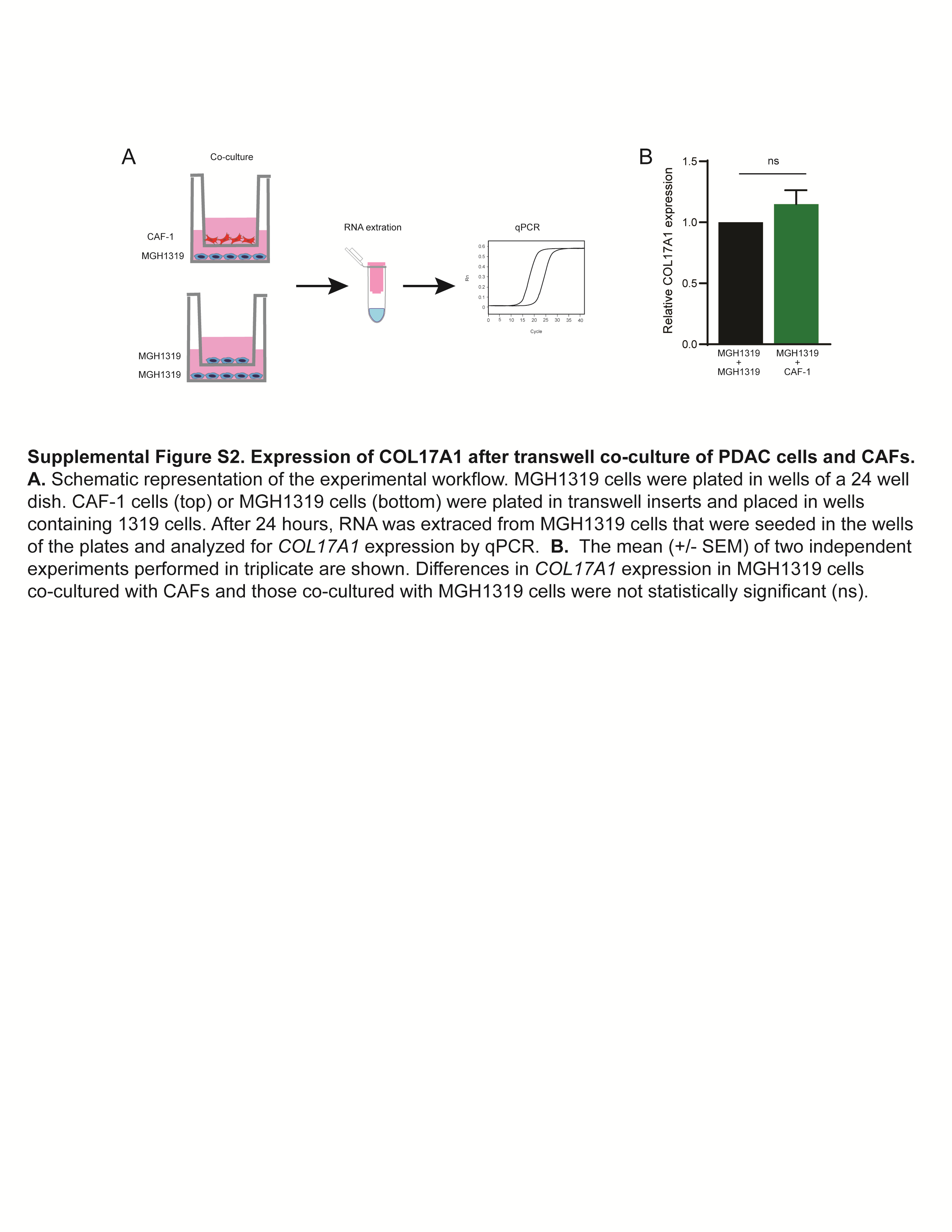

Supplement: Supplemental Figure S2 — Expression of COL17A1 after Trasnwell co-culture of PDAC cells and CAFs [file crc-24-0392_supplemental_figure_s2_suppsf2.png]

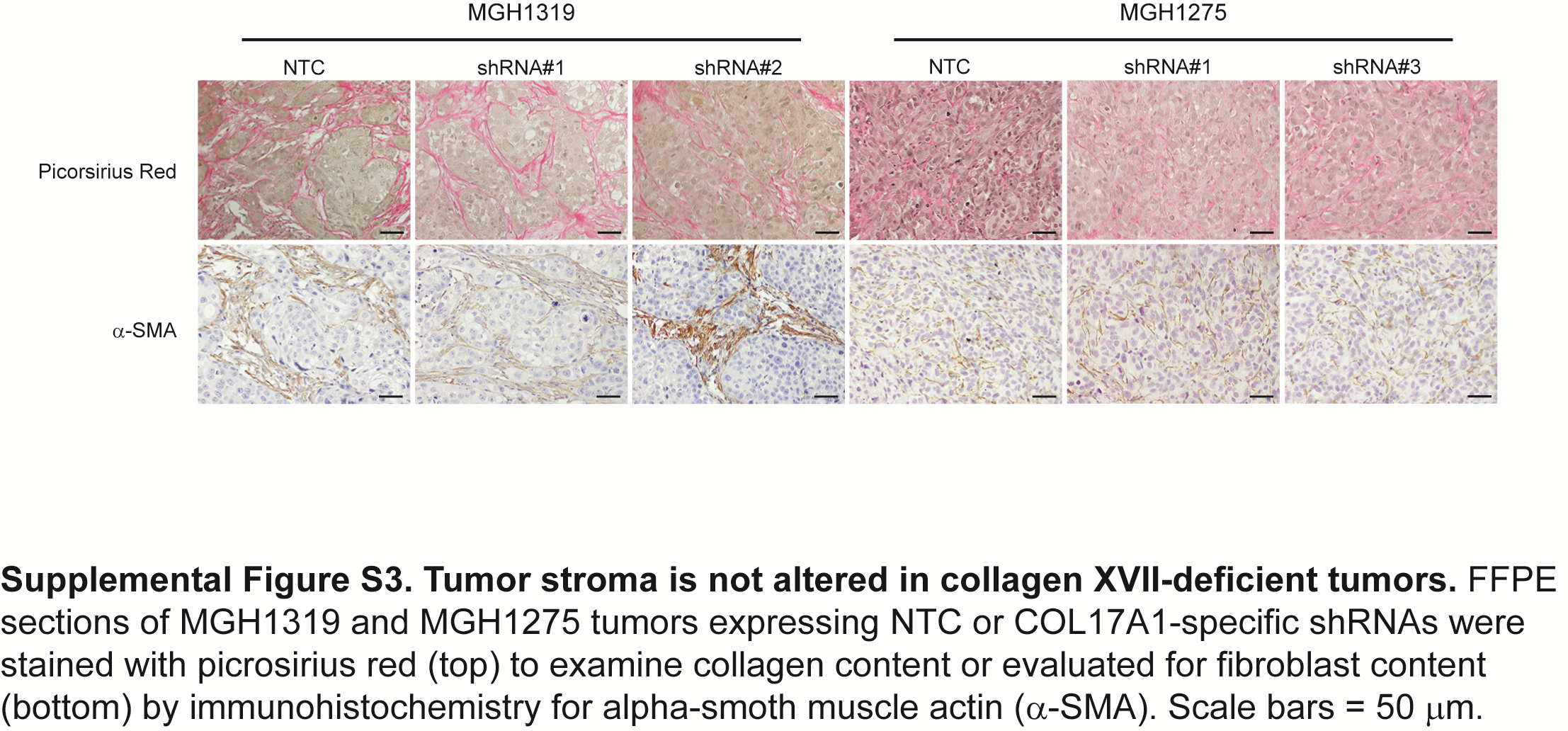

Supplement: Supplemental Figure S3 — Immunohistochemical and histologic analysis of collagen and fibroblast content of tumors formed by COLXVII-deficient PDAC cells [file crc-24-0392_supplemental_figure_s3_suppsf3.png]

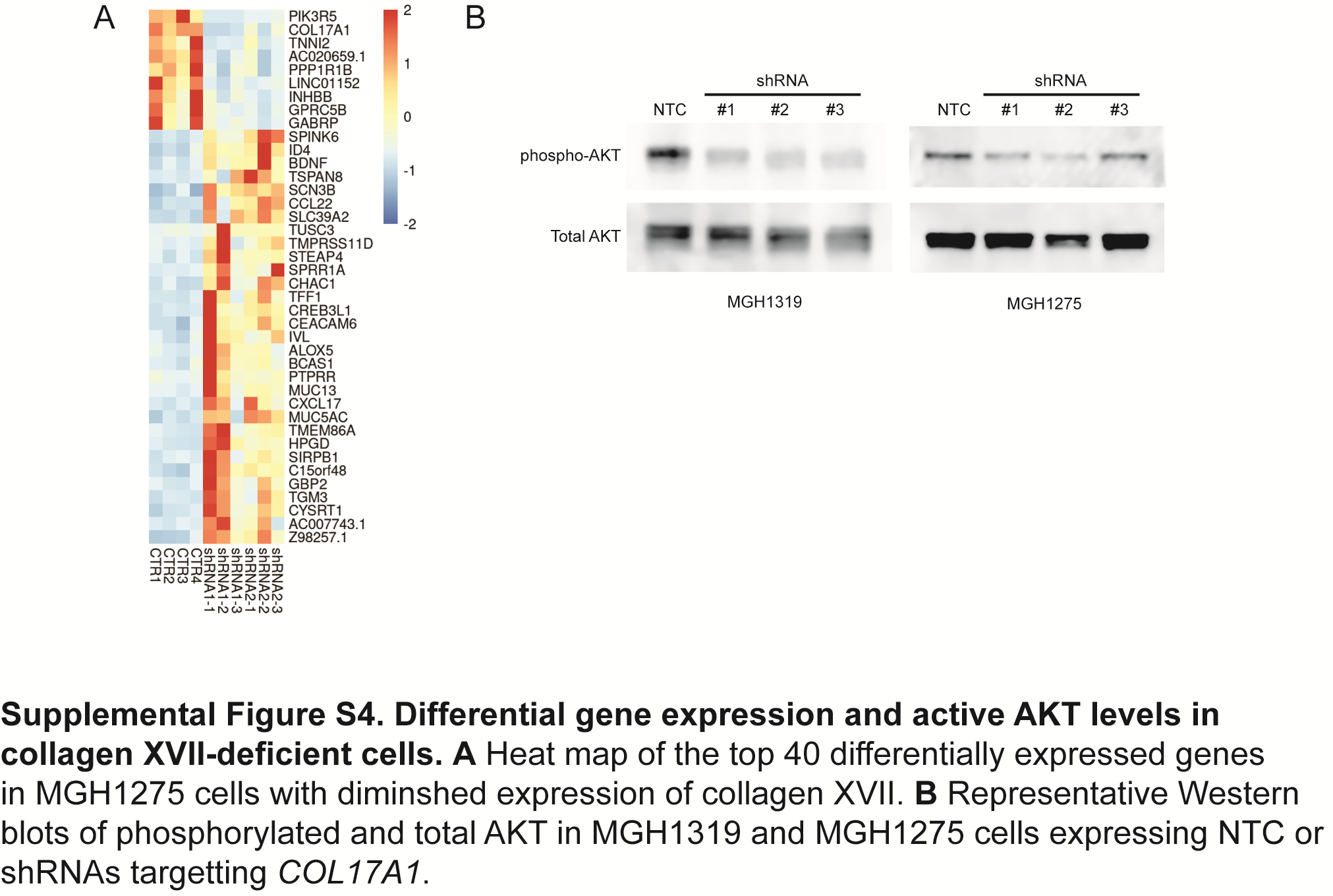

Supplement: Supplemental Figure S4 — Changes in gene expression and active AKT levels in collagen XVII-deficient cells [file crc-24-0392_supplemental_figure_s4_suppsf4.png]
